# Supplementary figures and images for: A propidium iodide-based in vitro screen of the “Bug Box” against Babesia duncani reveals potent inhibitors
Source: Antimicrob Agents Chemother. 2025 Jun 9;69(7):e00035-25. doi: 10.1128/aac.00035-25 (PMC12217462; doi:10.1128/aac.00035-25)

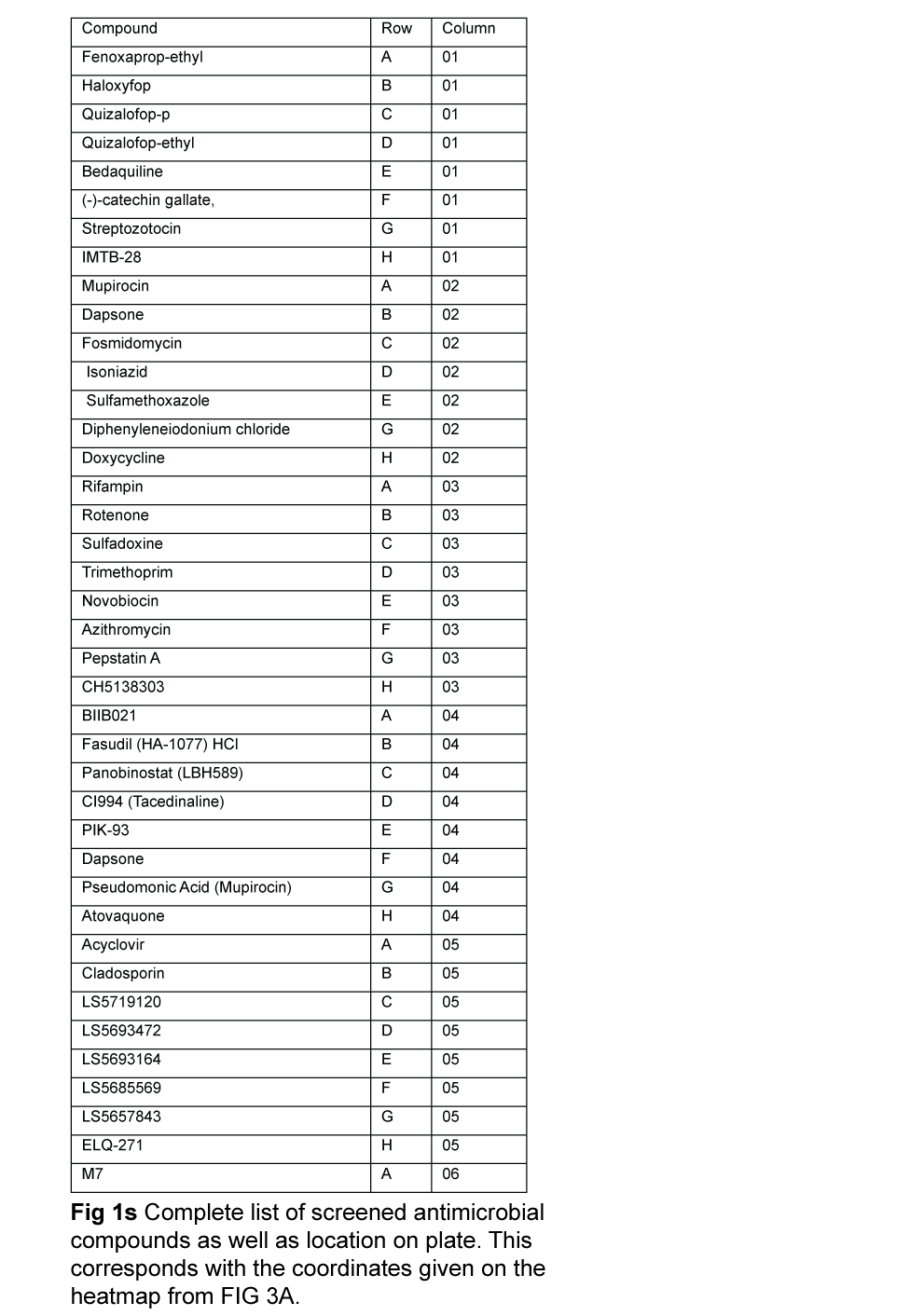

Supplement: Fig. S1 — Screened compounds from "Bug Box". [file aac.00035-25-s0001.tif]
